# Efficacy of the Sub-Urethral Transobturator KIM System^®^ for Female Urinary Incontinence: Long Term Results

**DOI:** 10.3390/jcm13195728

**Published:** 2024-09-26

**Authors:** María-Fernanda Lorenzo-Gómez, Javier-Antonio Flores-Carvajal, Magaly-Teresa Márquez-Sánchez, Gerardo-Alfonso Márquez-Sánchez, Javier Flores-Fraile, Filipa-María Alves-Rodrigues, Jose-Antonio Miron-Canelo, Bárbara Yolanda Padilla-Fernández

**Affiliations:** 1 Department of Surgery, University of Salamanca, 37007 Salamanca, Spain; 2 Group GRUMUR (Urology Multidisciplinary Research Group), IBSAL (Institute for Biomedical Research of Salamanca), 37007 Salamanca, Spain; magalymarquez77@gmail.com (M.-T.M.-S.);; 3 Department of Urology, University Hospital of Salamanca, 37007 Salamanca, Spain; 4 Department of Urology, Hospital of Specialties of the Honduran, Institute of Social Security, Tegucigalpa 11101, Honduras; 5 Department of Preventive Medicine and Public Health, University of Salamanca, 37008 Salamanca, Spain; 6 Urology Section, Department of Surgery, University La Laguna, 38200 Tenerife, Spain

**Keywords:** stress urinary incontinence, KIM^®^ system, SUI in women, SUI treatment, urethral transobturator

## Abstract

**Background/Objectives:** Female stress urinary incontinence (SUI) surgical treatment has changed since its beginning. Selecting materials that promote constructive tissue remodelling helps to maintain continence after surgery and minimizes complications. To analyze the long-term urinary continence results in women who underwent SUI surgical correction using the transobturator mid-urethral sling KIM system^®^ (Knotless Incontinence Mesh) and analyze the complications associated with this SUI treatment. **Materials and Methods:** Prospective study of the first 1000 patients undergoing SUI surgery with the Contasure KIM^®^ sling between April 2007 and December 2018. Results and complications were recorded. Group A represented 94.2% of the sample and were the continent patients after surgery (GA = 942), while Group B accounted for incontinent patients after surgery (5.8%) (GB = 58). **Results**: The mean age at operation was 60 years. Eutocic deliveries (*p* = 0.0022), high blood pressure (*p* = 0.0190), anxiety (*p* = 0.0084), hemorrhoidectomies (*p* = 0.0016) and hysterectomies (*p* = 0.0002) were higher in GB. No differences between groups were found regarding body mass index (GA 26.50; GB 26.93) (*p* = 0.220649), food or drug allergies (*p* = 0.0.6547), dystocia (*p* = 0.2365), diabetes mellitus (*p* = 0.1715), pelvic surgery (*p* = 0.8842), other surgery (*p* = 0.8801) or concomitant treatments that would have an impact on bladder function. Correction of SUI was achieved in 94.2% of cases. Continence persisted over time in 97.98% of continent patients at 4-year follow-up and 90.90% of patients at last follow-up. Mesh caused erosion of the urethra in 0.3% of patients and extruded to the vagina in 0.42%. De novo urinary urgency (2.8%), significant chronic pain (3.6%) and urinary tract infections (3.0%) after surgery were lower than complications reported in reviewed publications. Pain was treated with medication, and all patients were pain-free at the one-year follow-up visit. **Conclusions**: The mid-urethral transobturator sling KIM system^®^ has good initial and long-term results in patients with stress urinary incontinence, with a low recurrence rate and minimal complications.

## 1. Introduction

Any involuntary loss of urine affecting a patient is considered urinary incontinence [1]. It is a worldwide problem with 18.3 million cases in the US in 2010 and 28.4 million predicted by 2050. In Europe, its prevalence is higher in those aged 45 to 59 years [1]. In Spain, it is more frequent in people older than 60 and 64 years (40% and 35.1%), with rates of 23% in women over 18 years, 20% in working-age women, and 14% among women aged 40 to 64 [2].

Female stress urinary incontinence (SUI) surgical treatment has changed since its beginning. In 1990, Delancey emphasized the critical role of the sub-urethral area in achieving success in SUI surgical treatment [3]. Ulmsten and Petros reported on the retropubic tension-free vaginal tape (TVT) in 1995 [4,5]. In 2001, Delorme published about the transobturator technique (TOT): positioning a mesh behind the mid-urethra through the obturator foramen [6].

These innovations made the surgical treatment of stress urinary incontinence easier by enabling a less invasive approach. While managing uncomplicated stress urinary incontinence, the mid-urethral slings are the most frequent surgical option used [7].

Selecting materials that promote constructive tissue remodelling helps to maintain continence after surgery and minimizes complications [8].

This study aims to report on the long-term urinary continence results in a sample of women who had SUI corrective surgery with the transobturator mid-urethral sling KIM system^®^ (Knotless Incontinence Mesh), as well as the complications associated with this SUI treatment.

Female stress urinary incontinence treatment involves different steps for resolution: diagnosis, selection of the treatment, implementation of the treatment, and long-term performance of the chosen treatment. In this study, we will isolate the diagnosis, selection of treatment, and treatment implementation, as these factors depend on the medical decision and skills, and we will analyze the long-term performance of the KIM system^®^ once successfully implanted.

## 2. Materials and Methods

A prospective, multicentric study was performed with a sample of 1000 patients diagnosed with SUI who had TOT implantation with the mid-urethral sling KIM system^®^ (Neomedic International©, Terrassa, Spain) as treatment between April 2007 and December 2018 at the University Hospital of Salamanca and the Hospital Santisima Trinidad of Salamanca. 

The Clinical Research Ethics Committee of the University Hospital of Salamanca evaluated and approved the study with CEIC reference E.O. 12/274. All patients treated in that period were included, excluding patients who received either concomitant surgical treatment or another kind of mesh. Given the nature of the procedure, with programmed appointments for follow-up, all patients included in this study had follow-up data available for at least 4 years postoperatively.

The standard study protocol included taking a medical history, general and urogynecological physical exams, the validated Spanish version of the ICIQ-SF incontinence questionnaire [9], routine laboratory tests, and urine culture, and ultrasonography images of the kidneys and bladder were analyzed at 48 h, 3 months and yearly thereafter. Additionally, following standard practice, cystography, urodynamic tests, urine cytology, and cystoscopy were carried out as needed.

### Surgical Procedure

**Figure 1:**
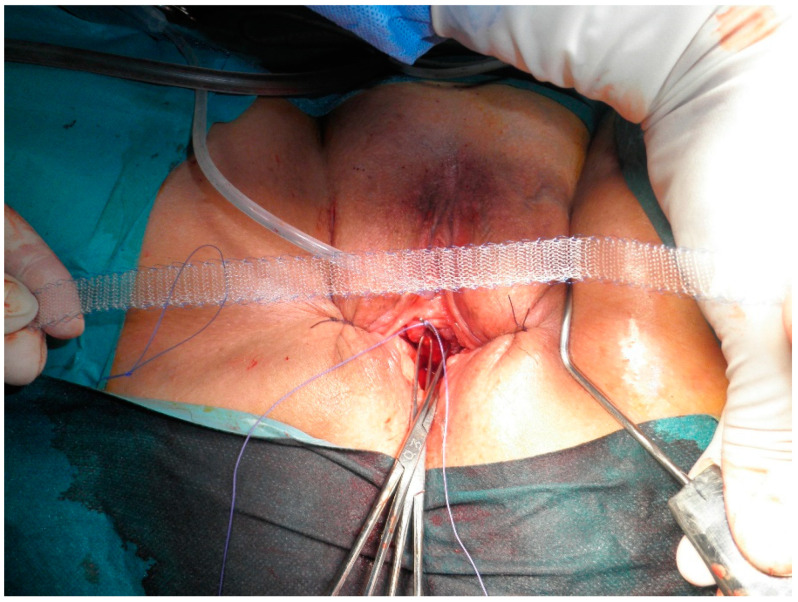
Insertion of outside-in, fine helical atraumatic needle. Source: Dra. María Fernanda Lorenzo Gómez’s file, Head of Urology Department of University Hospital of Salamanca.

All procedures were carried out requiring only one night of hospitalization in the short-stay unit using spinal anesthesia and antibiotic prophylaxis. The patient was positioned in the dorsal lithotomy posture with thighs in hyper-abduction. The bladder was catheterized using a 16 Ch ballon catheter fixed with 20 mL in the balloon. Hydro dissection was performed between the urethra and the anterior vaginal wall. Fine helical atraumatic needles, specifically designed for the procedure, were inserted from the outside (Figure 1). The tape was connected to the needle and passed inside out. Finally, a vaginal tamponade was placed for 12–24 h and removed before discharge, while the catheter was taken out 24 h after the procedure.

Continent patients were those with a score of 0 on the validated Spanish version of the ICIQ-SF incontinence questionnaire [9]. Incontinence was defined as a score of 1 or more on the questionnaire.

Every patient had an anamnesis to gather information regarding age and secondary diagnoses; they also had general and urogynecological physical exams, routine laboratory tests, urine culture, and ultrasonography of the kidneys and bladder, and answered the ICIQ-SF questionnaire.

We recorded effectiveness and complications, which included extrusion to the urethra, extrusion to other organs, extrusion to the vagina, infection, pain, urinary retention (either impossible urination or post-voiding urine volume greater than 150 cc), and the development of new urinary incontinence; pelvic surgery included surgical procedures for genital prolapse, cystocele, rectocele, and perianal fistula. Hysterectomy was considered a surgical resection of the uterus with or without adnexectomy.

Statistical analysis: We used descriptive statistics, Fisher’s exact test, and Student’s *t*-test. A *p*-value < 0.05 was considered statistically significant.

## 3. Results

After surgery, we distinguished two groups: Group A (GA = 942), 94.2% continent patients after surgery, and Group B (GB = 58), 5.8% incontinent patients after surgery. Group A was divided into two subgroups: GA1 = 909: patients who maintained continence at the last follow-up; GA2 = 33: patients who were not continent at the last follow-up.

The overall median follow-up time was 7.22 years, SD 2.27 years, median 7 years, and range 4–15 years. The follow-up time was shorter in GB compared to GA (*p* = <0.050) and in GA2 (6.50 years) compared to GA1 (7.39 years) (*p* = 0.000068).

**Figure 2:**
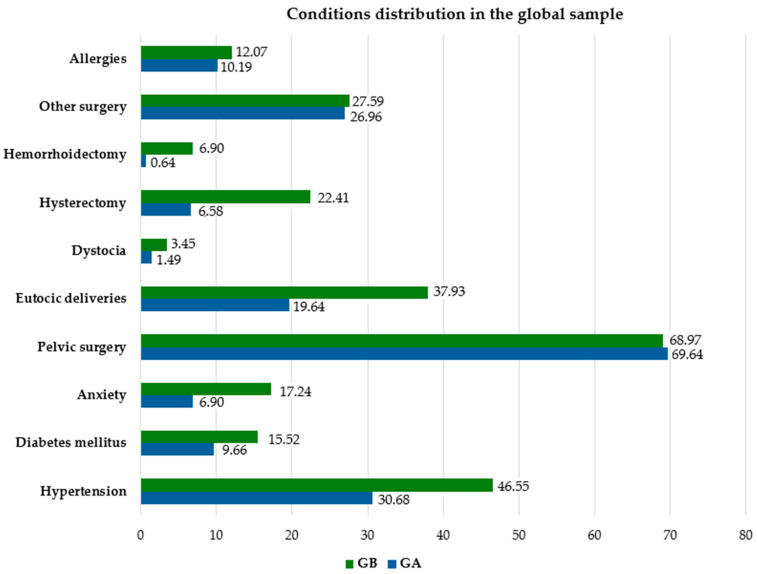
Conditions (%) in GA and GB.

In Group B, the average age (64.93 years) was higher than in Group A (59.36 years) (*p* = <0.050). There were more eutocic deliveries in GB than in GA (*p* = 0.0022), higher blood pressure in GB than in GA (*p* = 0.0190), more anxiety in GB than GA (*p* = 0.0084), more hemorrhoidectomies in GB than GA (*p* = 0.0016), and more hysterectomies in GB than in GA (*p* = 0.0002). There were no differences in body mass index between the groups (*p* ≥ 0.050), food or drug allergies (*p* = 0.6547), dystocia (*p* = 0.2365), diabetes mellitus (*p* = 0.1715), pelvic surgery (*p* = 0.8842), other surgery (*p* = 0.8801) or concomitant treatments that would have an impact on bladder function (Table 1, Figure 2).

Of the 1000 patients analyzed, initial continence (score 0) was achieved in 94.20% of the cases (GA). Persistent SUI (score ≥ 1) was found in 5.80% (GB). De novo urgency UI was found in 2.8% (6 patients from GA2 and 22 from GB, none from GA1). At the 4-year follow-up, 97.98% (GA1) continued to be continent and 2.01% were incontinent (GA2) in GA, and 37.93% were continent in GB. At the last follow-up, 90.9% scored 0 in GA1 and 3.30% scored ≥1 in GA2.

Complications were analyzed during the follow-up. We found urethral extrusion of the sling (Clavien–Dindo III [10]), overall 0.30%, in Group B compared to none in Group A (*p* = 0.0002); these extrusions were diagnosed at 6, 9 and 12 months of follow-up, respectively. These patients had stress urinary incontinence. All meshes were completely removed, and the reconstruction of the ventral urethra was performed with Vicryl 2.0, in “U” continuous transverse stitches, from right to left through the mucosa, to secure the submucosal tissue and prevent erosion of the urethra.

Extrusion towards the vagina (Clavien–Dindo III [10]) was observed in 0.42% in GA2 compared to none in GB (*p* = 0.0153) and none in GA1 (*p* = 0.0001); these extrusions were diagnosed at the 0,5-, 1-, 2-, and 2-year follow-ups, respectively. These patients were continent until the meshes were removed. After mesh explantation, they all presented stress urinary incontinence.

Urinary tract infection was seen in 3.0% and diagnosed at a mean of 90 days after surgery (SD 35.21, median 85, rank 21–180). All urinary infection cases (Clavien–Dindo II [10]) were solved with conventional antibiotic treatment, all were in GB and none in GA (*p* = 0.0001), and all were patients with an antibiotic allergy that did not allow optimal prophylaxis coverage, patients with long-evolution insulin-dependent diabetes, or smokers of more than 20 cigarettes a day.

Significant chronic pain lasting more than a month after surgery was observed in 3.6%; all were treated with medication (Clavien–Dindo I [10]), and all of them were cured at the one-year follow-up visit (Table 2). Pain was more frequent in GB than in GA (*p* = 0.0001), and within GA, it was more frequent in GA2 than in GA1 (*p* = 0.0001). De novo urgency was observed in 28 patients in total (2.8%): 6 cases (18.18%) in Group A2 and 22 cases (37.93%) in Group B (*p* = 0.0608).

## 4. Discussion

The goal of the SUI surgical intervention is to provide the right urethral support to avoid urine leakage under stress. Suture techniques like Kelly (1913), Stockel (1924) and Marion (1935) were used initially. In our field, we have mainly used the classic techniques of the suprapubic colposuspension of Marshall–Marchetti–Krantz (1949) and its Burch (1961) variant modification.

From 1996 to 2003, the TVT technique was used as a first option. Since 2003, TOT has been introduced to eliminate the passage through the retropubic space. Salo et al. have recently published a comparison between these techniques in 72 patients with a median follow-up time of 16 years where they observed that the severity of urinary incontinence (UI) was reduced significantly at 3-month follow-up. Furthermore, at the 16-year follow-up, UI severity, measured by the Urinary Incontinence Severity Score (UISS), was higher than at the 3-month follow-up but, when compared to baseline results, it was significantly lower, presenting successful long-term results with both techniques. However, the study did not account for complications [11].

Other studies have shown TOT to have a lower risk of vascular complications, intestinal injury or bladder perforation [12,13], but there is still some risk of harming the vessels and the obturator nerve with the passage of the needle. Although it is unlikely, cases of bladder perforation have also been described, especially when there is bladder prolapse [14].

In the classic technique, a Pfannestiel suprapubic incision is made, and the patient is released from the hospital at an average of 5 days. In contrast, with the TOT procedure, the patient only stays overnight at the hospital. It is even possible to perform the technique as an outpatient procedure.

Though this represents great savings for the health system, this would not be efficient in the case of recurrent incontinence, or if the prosthetic materials cause rejection or other complications (infection, migration, decubitus, ulcer, etc.) in the long term, thus requiring repeat intervention. Therefore, it is pertinent to investigate the long-term results in urinary continence and the evolution that these prostheses present.

A mesh should not induce an inflammatory reaction, an allergy or tumour [15]. In the case of a sub-urethral mesh, an exaggerated inflammatory reaction of the patient could potentially set off a chain of adverse reactions such as inducing an enzymatic degradation of the mesh, which could produce mechanical failure, or fibrotic process due to chronic inflammation that would end up producing erosion of native tissues [8].

Furthermore, when mesh incorporation into the surrounding tissues of the patient is slow or prevents the growth of fibrous and vascular structures through its pores, as seen with microporous or high-density meshes, it contributes to dead spaces forming between the prostheses and the patient’s tissues, which can lead to seroma and infection [15].

Consequently, what the mesh is made of and its characteristics (biocompatibility, how it is knitted, elasticity, flexibility, stability, elongation, pore size) can predispose to infection, especially in those with a pore size of less than 75 microns [8,16].

The surgeon has an important impact on the diagnosis, the elected treatment and the implantation technique. Short-term results will be more related to the surgeon’s experience, while long-term results will be more dependent on the performance of the implanted material. If there is no additional surgical procedure, the surgeon will not have an impact on the mesh’s long-term performance.

It is known that continence results decrease in time, with short-term continence being over 90% but long-term results at 80% or even below [17,18,19]. Croghan et al. [20] published a long-term outcome study of transobturator tape in Ireland with a mean follow-up time of 9.25 years in 2020; they observed a cure rate (patient-reported status of being almost entirely continent) of clinical stress urinary incontinence of 91% at 3-month follow-up, which lowered significantly to 59% at 9-year follow-up.

Our findings showed a similar follow-up time with a mean of 9.44 years, ranging between 4 and 15 years, which was greater in the GA group than in the GB group. In a study carried out by Abdel-Fattah et al., they compared two approaches of tape insertion while managing stress urinary incontinence with transobturator tapes (inside-out versus outside-in) with results after a year reporting an 80% success rate, similar between both groups (‘outside-in’ 77.6% versus ‘inside-out’ 81.2%) [21], with a significant drop in the patient-reported success rate at 3-year follow-up (*p* = 0.005) (‘outside-in’ 72.3% versus ‘inside-out’ 73.2%) [18], whereas in our study, the group with the longest follow-up time was the most successful with significant differences (*p* ≤ 0.050).

Our study shows, in contrast, the knotless KIM System^®^ mid-urethral sling with overlocked edges and no elongation (no deformation with traction), which achieves good continence results (94.2%), with 97% of these patients maintaining continence at the last follow-up (4–15 years), which demonstrates the efficacy, safety and reliability of the implanted material. Many slings used for urinary stress incontinence treatment do elongate under traction. The fact that the KIM system does not elongate may play an important role in maintaining long-term results.

The most common sling complications are exposure, extrusion, pain and infection. At the end of their study, Croghan et al. found only early complications, with acute urinary retention as the most common of them (6%), followed by urinary tract infection (4%) and pain (4%) [20]. In our study, we found similar results, with pain as the most common complication (3.6%), followed by urinary tract infection (3.0%) and de novo urgency (2.8%). 

In the study by Croghan et al., the Monarc^®^ subfascial hammock (AMS) tape was used; one of the characteristics of this mesh is that it can be adjusted after the primary surgery, but this is achieved by a suture threaded into the mesh [20]; meanwhile, the KIM system^®^ (Knotless Incontinence Mesh) used in our study has no knots and therefore has no spaces where bacteria may be protected from macrophages. The low extrusion (0.4%) and erosion (0.3%) rates that we see in this study may also be related to the absence of knots, which not only eliminates the presence of micro spaces but also minimizes the profile of the mesh and the friction with surrounding tissues.

In the European clinical guidelines, a Cochrane meta-analysis of 55 studies was reported where they compared the insertion of a sub-urethral device through the retropubic (TVT) or transobturator route (TOT), without finding significant differences in the cure rates at 12 months (71–97% and 62–98%, respectively). After TOT, there was less voiding dysfunction (4%) and risk of bladder perforation (0.2%) compared to TVT insertion (7% and 5%, respectively) [7]. Twenty-one of the trials reported chronic pain 12 months after surgery, while de novo urgency was reported in 6% of cases and vaginal perforation in 1.7% [22]; our study shows similar results to previous publications regarding de novo urgency urinary incontinence after tape insertion (10.6%), though it is worth noting that when continence was achieved after surgery, the probability of developing urgency urinary incontinence was 0.60%.

In the latest COCHRANE review, the medium- to long-term results were analyzed; after surgery, there were no significant differences in subjective healing when comparing TOT vs. TVT (RR 0.95, 95% CI 0.80–1.12). TOT reported 82% to 91% of patients being cured, while TVT showed a similar range (77% to 98%) [12]. In this study, the effectiveness of Kim System^®^-type transobturator sub-urethral tapes (Neomedic©) to correct stress urinary incontinence was within that range (87%).

Thus, while treating stress urinary incontinence, careful patient diagnosis, treatment indication, and technique performance are fundamental; the selection of mesh to maintain long-term results and minimize complications is equally important.

Furthermore, considering that our surgical team has extensive experience in the Marshall–Marchetti–Krantz technique, it is essential to conduct future comparative studies that evaluate the effectiveness and safety of the KIM^®^ transobturator system compared to other surgical techniques for the treatment of urinary incontinence, although our study, by comparing homogeneous groups, minimizes bias. The reported intraoperative complications (only two cases) were resolved conservatively and limited the possibility of making an exhaustive comparison of this aspect.

## 5. Conclusions

The mid-urethral transobturator sling KIM system^®^ has good initial and long-term results in patients with stress urinary incontinence, with a low recurrence rate and minimal complications.

## Figures and Tables

**Table 1:** Conditions in GA and GB, Student’s *t*-test and Fisher’s exact test.

Variables		Mean	SD	Median	Range		*p*-Value
Follow-up time (years)	GA	7.28	±2.27	7	4–15		0.032210
	GB	6.82	2.25	6	4–12		
Age (years)	GA	59.36	±13.45	59	20–87		0.000009
	GB	64.93	±11.77	68	35–87		
BMI (kg/m^2^)	GA	26.50	±4.53	25.78	17.96–50.78		0.220649
	GB	26.93	±4.51	26.43	18.36–37.10		
**Groups**	**Group GA, n = 942**		**Group GB, n = 58**		**Total, n = 1000**		***p*-Value**
Variables	n	%	n	%	n	%	
Hypertension	289	30.68	27	46.55	316	31.6	0.0190
Diabetes mellitus	91	9.66	9	15.52	100	10	0.1715
Anxiety	65	6.90	10	17.24	75	7.5	0.0084
Pelvic surgery	656	69.64	40	68.97	696	69.6	0.8842
Eutocic deliveries	185	19.64	22	37.93	207	20.7	0.0022
Dystocia	14	1.49	2	3.45	16	1.6	0.2365
Hysterectomy	62	6.58	13	22.41	75	7.5	0.0002
Hemorrhoidectomy	6	0.64	4	6.90	10	1	0.0016
Other surgery	254	26.96	16	27.59	270	27	0.8801
Allergies	96	10.19	7	12.07	103	10.3	0.6547

**Table 2:** Clavien–Dindo classification and complications, Fisher’s exact test.

Groups		Group GA, n = 942		Group GB, n = 58		Total, n = 1000		*p*-Value
Clavien–Dindo clasification	Complications	n	%	n	%	n	%	
Grade I	Chronic pain	22	2.34	14	24.14	36	3.60	0.0001
Grade II	Urinary tract infection	0	0.00	30	51.72	30	3.00	0.0001
Grade II	Urethral extrusion of the sling	0	0.00	3	5.17	3	0.30	0.0002
	Extrusion towards the vagina	4	0.42	0	0.00	4	0.40	1.0000
**Subgroups**		**Group GA1, n = 909**		**Group GA2, n = 33**		**Total, n = 942**		***p*-Value**
Clavien–Dindo clasification	Complications	n	%	n	%	n	%	
Grade I	Chronic pain	10	1.10	12	36.36	22	2.34	0.0001
Grade II	Urinary tract infection	0	0.00	0	0.00	0	0.00	1.0000
Grade III	Extrusion towards the vagina	0	0.00	4	12.12	4	0.42	0.0001

## Data Availability

The original contributions presented in the study are included in the article, further inquiries can be directed to the corresponding authors.
